# Overestimation of driving pressure by the analysis of the conductive pressure during venous-arterial ECMO: Airway Closure or Intrinsic PEEP?

**DOI:** 10.1186/s13054-023-04772-4

**Published:** 2023-12-21

**Authors:** Emanuele Rezoagli, Matteo Pozzi, Maurizio Cereda, Giuseppe Foti

**Affiliations:** 1 School of Medicine and Surgery, University of Milano-Bicocca, Monza, Italy; 2 Department of Emergency and Intensive Care, Fondazione IRCCS San Gerardo dei Tintori, Monza, Italy; 3 Department of Anesthesia, Critical Care and Pain Medicine, Massachusetts General Hospital, Boston, MA USA; 4 Harvard Medical School, Boston, MA USA

Driving pressure (DP) is the elastic distending pressure of the respiratory system. High DP promotes lung stress [1]. A reliable estimation of DP is crucial to prevent lung injury during mechanical ventilation. DP is measured during tidal ventilation in volume-controlled mode as the difference between plateau and positive end-expiratory pressure (PEEP) (i.e., apparent DP). However, the presence of airway closure (AC) may affect the reliability of DP estimation in patients with both lesional [2] and hydrostatic [3] pulmonary edema, explaining the difference between apparent and actual DP. In a recent issue of *Critical Care*, Haudebourg AF. and colleagues proposed an elegant measurement of the conductive pressure during tidal ventilation (i.e., Pcond) as a valuable tool to evaluate whether apparent DP overestimates actual DP because of AC in critically ill patients undergoing mechanical ventilation [4].

In patients without AC, Pcond is equal to the resistive airway pressure (Pres = Peak and plateau pressure) after an end-inspiratory hold maneuver. As shown in Fig. 1, panel A, the apparent DP matches the actual DP as Pcond = Pres.

**Fig. 1:**
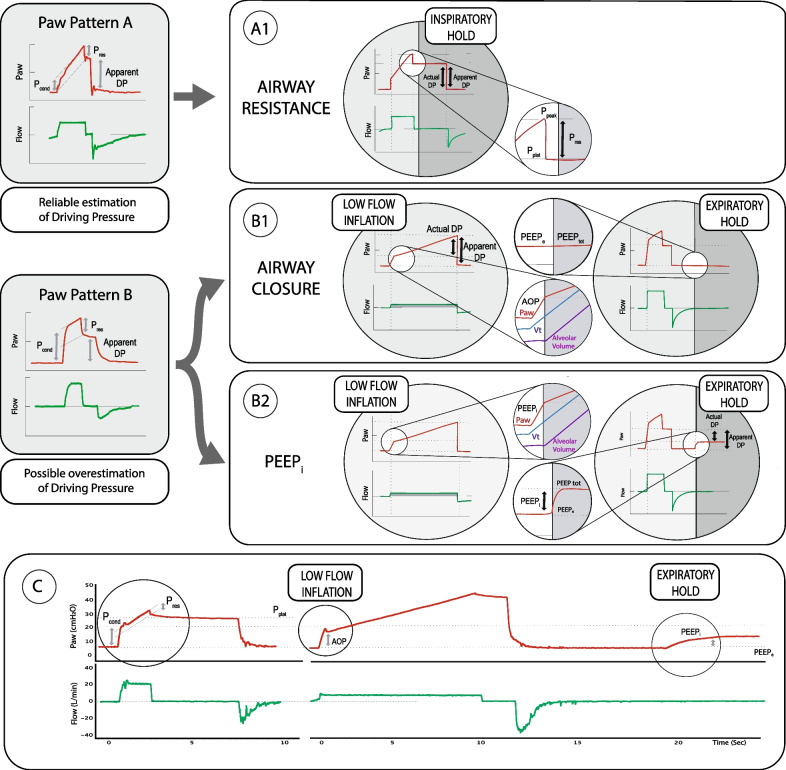
Patterns of conductive pressure–time waveform (Pcond) during volume-controlled ventilation. In pattern A1, the apparent DP matches the actual DP as Pcond = Pres. In pattern B1, respiratory pattern with AC where Pcond may unveil DP overestimation because Pcond > Pres. AC can be unveiled by a low-flow inflation pressure–time curve with a flow of 5 L/min. In pattern B2, respiratory pattern with intrinsic positive end-expiratory pressure (i.e. PEEPi) where Pcond may unveil DP overestimation because Pcond > Pres. PEEPi can be detected by an end-expiratory hold maneuver. In panel C, patient undergoing venous-arterial ECMO for cardiac arrest and severe respiratory failure with the presence of both phenomena leading to DP overestimation (i.e., AC and PEEPi) that can be visually detected by the presence of Pcond as first (on the left), and that can be subsequently quantitatively estimated by a low-flow inflation (in the middle) and by an end-expiratory hold maneuver (on the right). AOP, airway opening pressure with the visible “Uncorking effect”; DP, driving pressure; Paw, airway pressure; Pcond, conductive pressure; PEEPi, intrinsic PEEP; PEEPe, extrinsic PEEP; PEEPtot, total PEEP; Ppeak, peak pressure; Pplat, plateau pressure; Pres, resistive airway pressure; Vt, tidal volume

In contrast, in the presence of AC, Pcond unveils DP overestimation because Pcond > Pres. Subsequently, the presence of AC can be quantitatively investigated with a low-flow inflation pressure–time curve using a flow of 5 L/min as observed in Fig. 1, pattern B1, representing a patient with a respiratory pattern with AC.

However, Pcond may unveil DP overestimation also because of the presence of intrinsic positive end-expiratory pressure (i.e., PEEPi), although AC is not present. In this scenario, Pcond may be higher as compared to Pres because of the presence of PEEPi. PEEPi can be quantitatively estimated at bedside by an end-expiratory hold maneuver as in Fig. 1, pattern B2.

In this context, we here present the interesting application and interpretation of Pcond in the setting of extracorporeal support by venous-arterial ECMO in a patient presenting two different potential causes of airway closure: cardiac arrest—leading to hydrostatic pulmonary edema [3]—and severe respiratory failure—leading to lesional pulmonary edema [2]. At the visual inspection of Pcond, we captured a potential condition of DP overestimation. By applying both a low-flow inflation and an end-expiratory hold maneuver, we explained DP overestimation by both AC and PEEPi phenomena (Fig. 1, panel C).

Understanding the cause of DP overestimation in critically ill patients is pivotal as it may imply radically different therapeutic strategies. While AC may require optimization of PEEP titration, PEEPi may require the optimization of different ventilatory settings (e.g., tidal volume, respiratory rate, minute ventilation, and PEEP settings) and/or the administration of different pharmacological treatments (e.g., bronchodilators), according to its potential cause (e.g. dynamic hyperinflation, flow obstruction and flow limitation) [5].

## Data Availability

Not applicable.
